# *De novo* production of mandelic- and 3-phenyllactic acid from lignocellulosic feedstock through *Phlebia radiata* IBWF-87241

**DOI:** 10.1186/s12934-026-03159-8

**Published:** 2026-10-20

**Authors:** Antea Dulaj, Ida Malin Völlger, Joana Zoth, Emmanuel Akpan, Bernd Wetzel, Christiane Grünewald, Stefan Jacob, Luis Antelo, Eckhard Thines

**Affiliations:** 1 Johannes Gutenberg University Mainz, Mainz, Germany; 2 Institut für Biotechnologie und Wirkstoff-Forschung, Mainz, Germany; 3 Leibniz-Institut für Verbundwerkstoffe GmbH, Kaiserslautern, Germany

**Keywords:** *Phlebia radiata*, White-rot fungi, Mandelic acid, 3-phenyllactic acid, Aromatic α-hydroxy acids, Lignocellulose, Laccase, Bioproduction, GC–MS

## Abstract

**Background:**

*Phlebia radiata* is a white-rot basidiomycete that secretes a repertoire of oxidative enzymes making it a promising organism for the sustainable bio-production of aromatic compounds during lignocellulose-containing cultivation. Here, we investigate the association between wood supplementation and extracellular production of aromatic α-hydroxy acids by *P. radiata* IBWF 87241 under defined axenic conditions, while accompanying changes in extracellular oxidative enzyme activity and residual wood fibres were characterized using enzyme assays and ATR-FTIR spectroscopy.

**Results:**

GC–EI–MS analysis of culture filtrate extracts, confirmed by authentic standards, revealed two aromatic α-hydroxy acids produced exclusively under wood-containing conditions. Mandelic acid and 3-phenyllactic acid were produced by *P. radiata* in ½ soy medium supplemented with 1% (w/v) spruce wood powder, but were absent from un-supplemented and uninoculated controls across repeated cultivation experiments. Continuous monitoring of the cultivation shows that extracellular laccase activity increased throughout fermentation and reached a maximum on day 7, whereas H₂O₂-dependent ABTS oxidation remained low throughout the experiment, while esterase activity showed a minor transient increase. ATR-FTIR analysis of recovered wood powder revealed reproducible treatment-associated spectral differences relative to untreated controls in regions assigned to lignin- and carbohydrate-associated functional groups. Scale-up cultivation in a 20 L stirred-tank bioreactor yielded preliminary titers of 0.79 mg/L mandelic acid and 4.11 mg/L 3-phenyllactic acid, establishing baseline production levels under controlled process conditions.

**Conclusions:**

These findings establish *P. radiata* IBWF 87241 as a non-engineered fungal host for the production of aromatic α-hydroxy acids under wood-containing cultivation conditions and provide a quantitative baseline for future metabolic engineering and bioprocess optimisation strategies targeting mandelic acid and 3-phenyllactic acid production. To our knowledge, this is the first report of production of mandelic acid and 3-phenyllactic acid by *P. radiata* under defined cultivation conditions. The combined characterization of extracellular oxidative activity, FTIR spectral differences in recovered wood, and aromatic α-hydroxy acid production provides a framework for future investigations of extracellular oxidative metabolism, substrate-associated spectral alterations, and aromatic metabolite formation during fungal lignocellulose conversion.

**Graphical Abstract:**

**Figure:**
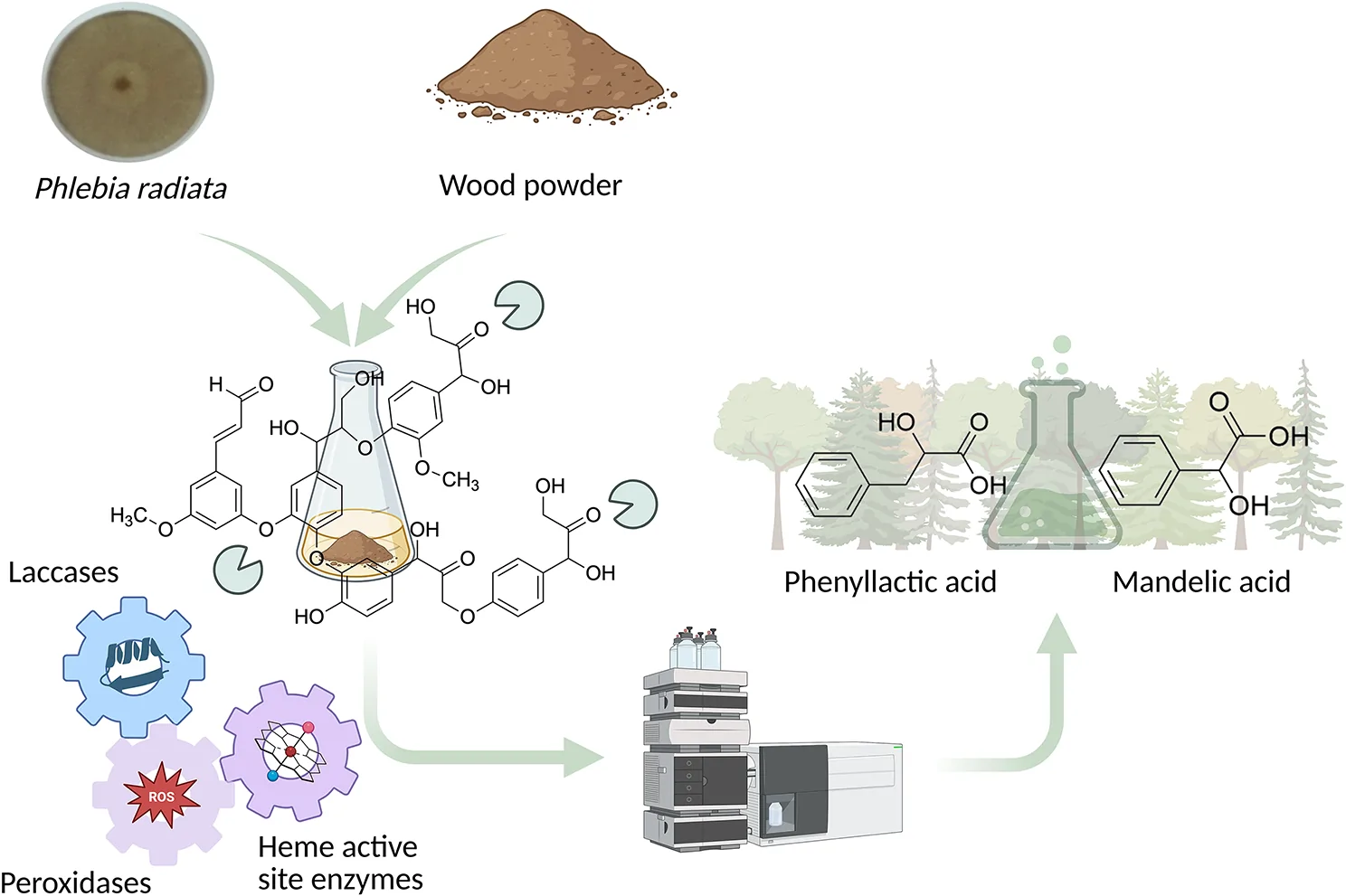

**Supplementary Information:**

The online version contains supplementary material available at https://doi.org/10.1186/s12934-026-03159-8.

## Introduction

Aromatic platform chemicals are highly demanded precursors for the manufacture of pharmaceuticals, polymers, coatings, and numerous other industrial products [1, 2]. Currently, the production of these platform chemicals is largely dependent on fossil carbon raw materials, motivating the need for the development of renewable alternatives. International initiatives such as the United Nations 2030 Agenda for Sustainable Development and the European Green Deal explicitly promote the transition toward circular, resource-efficient production systems based on renewable feedstocks and sustainable industrial processes [3, 4]. Several studies have shown that fungal metabolism of lignin-derived aromatics generates a variety of low-molecular-weight phenolics and related catabolic products. Under carefully controlled conditions, these processes can produce quantifiable amounts of platform chemicals that are scalable into commercial quantities, making way for a complete biotechnological means of developing these important platform chemicals.

Characterization of these extracellular metabolite profiles can provide valuable insight into the native capacity of fungal species to produce biotechnologically relevant aromatic compounds [5–7]. Oxidative modification of lignin can yield a chemically diverse set of soluble products, reflecting both the heterogeneity of the polymer and the reactions mediated by secreted enzymes [8–10]. Although genome analyses provide information on the enzymatic repertoires of ligninolytic fungi, they do not directly resolve which small molecules accumulate during wood degradation. Spatial metabolomics and multi-omics studies have revealed the presence and localization of lignin-derived compounds that cannot be inferred solely from gene content [11]. In addition, metabolomic profiling across fungal decay stages has identified hundreds of low-molecular-weight aromatic intermediates produced during wood decomposition that cannot be directly identified from genomic analyses alone [12, 13]. For this reason, parallel analysis of extracellular enzyme activity and culture metabolites has been widely applied to describe fungal wood conversion beyond bulk lignin modification [9, 10, 14].

Large amounts of wood processing residues remain underutilised despite their potential as renewable feedstocks for biomanufacturing. Activity on wood waste valorisation has increased substantially in recent years, with growing interdisciplinary engagement across environmental sciences, engineering, and bioenergy sectors [15]. Industrial implementation, however, remains dominated by thermochemical routes and low-value energy recovery. Wood is a lignocellulosic composite comprising cellulose microfibrils embedded in a hemicellulose-lignin matrix (35–50% cellulose, 20–35% hemicellulose, 15–30% lignin) [2]. Cellulose forms linear β-1,4-linked glucan chains assembled into crystalline microfibrils that provide structural rigidity [16]. Unlike the carbohydrate polymers cellulose and hemicellulose, lignin is a three-dimensional phenylpropanoid network derived from p-coumaryl-, coniferyl-, and sinapyl- alcohol units through oxidative coupling [16]. Its chemical heterogeneity and extensive cross-linking render depolymerization inherently challenging [8]. White-rot basidiomycetes secrete oxidoreductases, including laccases and peroxidases, that contribute to lignin modification, as part of their natural role in lignocellulosic biomass turnover. Fungal enzymes have therefore been investigated for the depolymerization of lignocellulose and for improving the accessibility of lignocellulosic biomass for subsequent conversion into biofuels and high-value biochemicals [17]. The ability of laccases to catalyze one-electron oxidation of phenolic substrates has driven continued interest in their application for sustainable lignocellulose conversion and the production of value-added biochemicals from renewable feedstocks [9, 14, 18]. Within the broader context of circular bioeconomy, there is increasing interest in fungi that convert lignocellulosic residues into higher-value products [12, 19].

*Phlebia radiata* is recognised as a robust ligninolytic fungus, and genome analysis reveals a large repertoire of carbohydrate-active enzymes (CAZymes) and auxiliary oxidoreductases (> 70 AA-class coding genes), consistent with oxidative enzyme involvement during wood conversion [12]. However, the low-molecular-weight metabolites produced during growth on lignocellulosic substrates remain incompletely characterised, particularly with respect to aromatic organic acids and related compounds. Understanding the formation of these metabolites is important for advancing fungal lignocellulose valorization and aromatic bioconversion strategies [19, 20]. Yet the capacity of *P. radiata* to accumulate specific aromatic target compounds at quantifiable titers under defined, scalable cultivation conditions has not been established, limiting its use as a non-engineered fungal production host for aromatic α-hydroxy acids.

The present study aimed to profile extracellular metabolites and characterize the associated extracellular enzyme activity dynamics and FTIR spectral profiles of recovered wood during lignocellulose-containing cultivation of *P. radiata* IBWF 87241. By integrating extracellular enzyme activity measurements, FTIR analysis of recovered wood, and metabolite profiling during bioreactor cultivation, we characterized these features in parallel within the same cultivation system. Under these conditions, mandelic acid and 3-phenyllactic acid were produced, establishing a quantitative baseline for aromatic α-hydroxy acid production in a non-engineered white-rot fungus and providing a foundation for future bioprocess development. To our knowledge, this represents the first report of these compounds in *P. radiata* IBWF 87241 under defined wood-containing cultivation conditions.

## Materials and methods

### Fungal preculture preparation

The *Phlebia radiata* IBWF 87241 strain was taken from the strain collection built and maintained by the Institut für Biotechnologie und Wirkstoff-Forschung (IBWF) gGmbH in Mainz, Germany. Precultures were grown in 100 mL Erlenmeyer flasks containing 50 mL of potato-dextrose broth (PDB) medium (Table S4). Flasks were inoculated with eight agar plugs from solid fungal cultures (PDA media). Precultures were incubated under shaking conditions at 23 °C until sufficient biomass was obtained for further use. Due to the exploratory nature of the study, metabolite extraction and analytical profiling were performed on a single representative culture per cultivation condition (*n* = 1) in each experiment. Small-scale cultivation experiments were conducted independently on two separate occasions, and the principal findings were subsequently confirmed in an independent 20 L stirred-tank cultivation.

### Cultivation conditions and sampling for extracellular enzyme activity and metabolite analysis

*P. radiata* IBWF 87241 was cultivated under sterile conditions in different liquid media, including minimal medium (MM), B5 medium, potato-dextrose broth (PDB), V8 medium, and ½ soy medium (½ SM), each cultivated with or without 1% wood powder (WP) (spruce) provided by Leibniz-Institut für Verbundwerkstoffe (IVW) (Table S4). Flasks were inoculated with 50 mL well-grown preculture and incubated at 23 °C under shaking conditions. For extracellular enzyme activity measurements, 2 mL culture samples were collected three times per week over an 18-day cultivation period. Samples were centrifuged and the supernatants used for extracellular enzyme assays. For metabolite profiling, 50 mL samples were collected twice per week over a 28-day cultivation period for downstream extraction and chromatographic analysis. For comparison, baseline samples collected prior to fungal inoculation, uninoculated medium controls, and uninoculated medium containing 1% (w/v) wood powder were included in the analytical workflow.

### Scale – up fermentation

Scale-up cultivation was performed in a 20 L stirred-tank bioreactor with a volume of 20 L. For bioreactor cultivation, a dedicated preculture was prepared in a 5 L Erlenmeyer flask containing 2 L of sterile ½ soy medium under the same cultivation conditions described above. After sufficient mycelial growth, the entire 2 L preculture was used to inoculate the bioreactor.

Cultivation was conducted at 23 °C under batch conditions with agitation at 150 rpm and aeration at 3 L/min. Antifoam (Sigma-Aldrich Chemie GmbH) was added as required. Culture samples of 50 mL were withdrawn on a daily basis for GC–MS analysis as described below. pH was measured using a calibrated SevenExcellence S400 pH meter (Mettler Toledo).

### Liquid-liquid extraction of natural compounds from fungal culture filtrate

Wood powder (WP) was separated by vacuum filtration from the broth; samples containing soy flour were centrifuged (7,800 rpm, 10 min) before filtration. Culture samples were mixed 1:1 with ethyl acetate (EA) and shaken briefly. The organic phase was filtered through sodium sulfate and concentrated using a rotary evaporator. Extracts were resuspended in 4 mL methanol, transferred to pre-weighed vials, and further concentrated in a vacuum concentrator until dry. Final vial weights were recorded to determine extract mass.

### Analytical HPLC and LC–MS Analysis with Electrospray Ionization (ESI)

Samples were prepared by resuspending dried insoluble extracts at 5 mg mL⁻¹ in methanol, followed by centrifugation (13,000 rpm, 10 min) to remove particulate matter. A 100 µL aliquot was transferred to HPLC vials for analysis. Analytical HPLC was performed on a Shimadzu LC system equipped with a reversed-phase C18 column (Waters SunFire C18, 4.6 × 250 mm, 5 μm) maintained at 20 °C. Separation was achieved using a linear gradient of aqueous 0.1% trifluoroacetic acid and acetonitrile at a flow rate of 1.0 mL min⁻¹ over 30 min with an injection volume of 20 µL. UV absorbance spectra were recorded between 200 and 800 nm using a diode array detector.

LC–MS analyses were performed on a Shimadzu LCMS-2020 single quadrupole system equipped with an electrospray ionization (ESI) source operating in both positive and negative ionization modes. Chromatographic separation was carried out on a Nucleoshell RP18 column (2 × 100 mm, 2.7 μm) maintained at 40 °C using a gradient of aqueous 0.1% formic acid and acetonitrile at a flow rate of 0.30 mL min⁻¹. The injection volume was 1–10 µL, and mass spectra were acquired over a scan range of m/z 150–1000. Source parameters included a desolvation line temperature of 250 °C, a nebulizing gas flow of 1.5 L min⁻¹, and a drying gas flow of 10 L min⁻¹ at 200 °C. HPLC–DAD data were processed using LabSolutions software version 5.99, and LC–MS data were processed using LabSolutions software version 5.109 (Shimadzu).

### Gas Chromatography – Electron Ionization – Mass Spectrometry (GC EI MS)

Dried extracts were resuspended in methanol at 5 mg/mL. An aliquot of 10 µL was evaporated to dryness and derivatized with 10 µL of 20 mg/mL ethoxyamine hydrochloride solution and 20 µL pyridine at 40 °C for 90 min. Subsequently, 70 µL N-methyl-N-(trimethylsilyl) trifluoroacetamide (MSTFA) was added and incubated for 50 min. Samples were centrifuged (13,000 rpm, 10 min), and 70 µL supernatant was transferred to vials for analysis. GC-MS analysis was performed using a Shimadzu GCMS QP 2010 equipped with an AOC20i autosampler and a DB5 MS capillary column (30 m × 250 μm I.D., 0.25 μm film thickness). Data and spectra were analyzed with the GCMS solution version 4.44. Helium was used as carrier gas at 1 mL/min. Injection volume was 1 µL in split mode (1:10) with the injector at 250 °C. The oven temperature was initially set to 70 °C, held for 5 min, then ramped at 10 °C/min to 320 °C and held for 10 min. Mass spectra were recorded in electron impact full scan mode (15–800 m/z) with an ion source at 200 °C and transfer line at 250 °C. Analyte identification was performed by comparison to the National Institute of Standards and Technology (NIST) mass spectral library.

For quantitative analysis, external calibration curves were generated from derivatized authentic standards of mandelic acid and 3-phenyllactic acid using five calibration levels with three technical injections per level. Peak areas at the quantification ion m/z 73 were fitted using an unweighted quadratic least-squares regression constrained through zero. The day-7 bioreactor extract was quantified from three technical GC–EI–MS injections; calibration data and calculation details are provided in Figs. S7–S8 and Tables S2–S3.

### Fourier Transform Infrared Spectroscopy (FTIR) measurement of wood powder

Following cultivation, the recovered wood powder was separated from the culture broth by filtration and washed thoroughly with ethanol followed by distilled water to remove residual medium components and extracellular metabolites. The washed material was subsequently dried at 45 °C and analysed by ATR–FTIR spectroscopy by placing the dried wood powder onto the ATR crystal surface.

FTIR measurements were performed using an IRXross Fourier transform infrared spectrophotometer (Shimadzu, Japan), equipped with a temperature-stabilized DLATGS detector, a ceramic infrared source, and a He–Ne laser for wavelength calibration. Spectra were acquired using a single-bounce diamond attenuated total reflectance (ATR) accessory (QATR-10, extended range), enabling measurements across a broad mid-infrared range with high signal-to-noise performance. All spectra were recorded in absorbance mode using 16 co-added scans at a spectral resolution of 4 cm⁻¹. Measurements were collected over the full mid-infrared range supported by the instrument (4000–400 cm⁻¹). Baseline correction was applied using asymmetric least squares (AsLS) baseline estimation (λ = 1 × 10⁶, *p* = 0.001, 10 iterations), and the estimated baseline was subtracted from each spectrum. Spectra were interpolated onto a common wavenumber grid with 2 cm⁻¹ spacing and vector-normalized (L2 normalization) prior to visualization as overlaid absorbance spectra with the wavenumber axis displayed in decreasing order.

### Qualitative plate-based screening of oxidative enzyme activity

Initial qualitative screening of ligninolytic enzyme activity was performed on agar plates prior to liquid cultivation [21]. Fungal isolates were inoculated onto lignin-modifying enzyme (LME) basal medium agar plates supplemented with indicator dyes. Plates were incubated in darkness at 23 °C for 10 days. Enzyme activity was inferred from color development (blue-green coloration of ABTS) or dye decolorization (Azure B and Poly R 478).

### Quantitative determination of extracellular laccase, peroxidase and esterase activity in liquid culture

Extracellular enzyme activity was quantified in liquid cultures using ABTS oxidation assays in 96-well microtiter plates. Culture filtrates (2 mL) were collected, centrifuged (13,000 rpm, 10 min), and assayed either undiluted or after dilution with deionized water (1:10, 1:100, or 1:1000). For laccase activity measurements, 100 µL of undiluted or diluted culture filtrate was mixed with 100 µL deionized water. For ABTS oxidation measurements in the presence of H₂O₂, 100 µL of diluted culture filtrate was mixed with 100 µL of a 4.5 mM H₂O₂ solution. In both cases, reactions were initiated by addition of 100 µL ABTS buffer (3.8 mM ABTS in 20 mM tartaric acid, pH 4.5). Absorbance at 420 nm was recorded continuously for 2.9 min using a FLUOstar^®^ Omega microplate reader. Esterase activity was measured photometrically at 405 nm by monitoring the hydrolysis of p-nitrophenyl acetate (PNP-A). A PNP-A stock solution was prepared by dissolving 0.0180 g PNP-A in 2 mL acetonitrile and diluting to 20 mL with 0.1 M sodium phosphate buffer (pH 8.0). The substrate solution was stored in amber vials at − 20 °C to prevent degradation. For the assay, 50 µL culture filtrate were mixed with 150 µL 0.1 M phosphate buffer (pH 8) and 50 µL PNP-A solution in a 96-well plate (total volume 250 µL). Blank controls contained buffer and substrate instead of culture filtrate, and heat-denatured enzyme controls were included to account for non-enzymatic hydrolysis. Absorbance at 405 nm was recorded over time immediately after substrate addition.

Enzyme activities were calculated as units per liter (U/L) based on the slope of absorbance change over time, taking into account total reaction volume, sample volume, dilution factor, the extinction coefficient of the ABTS radical cation at 420 nm (ε = 3.6 × 10⁴ L mol⁻¹ cm⁻¹) [22, 23], and optical path length. The optical path length (l = 0.38 cm) was estimated from the reaction volume (300 µL) and the cross-sectional area of a standard flat-bottom 96-well microplate according to (l = V/A). H₂O₂-dependent ABTS oxidation was calculated by subtracting ABTS oxidation measured in the absence of H₂O₂ from that measured in the presence of H₂O₂ and was used as an estimate of extracellular peroxidase activity. For esterase activity, the corresponding calculation was performed using the slope of the absorbance change at 405 nm and an extinction coefficient of ε = 18,000 M⁻¹ cm⁻¹ for p-nitrophenol. All measurements were performed in technical triplicates.

## Results

### Plate-based dye assays indicate extracellular oxidative enzyme activity in *Phlebia radiata*

**Fig. 1:**
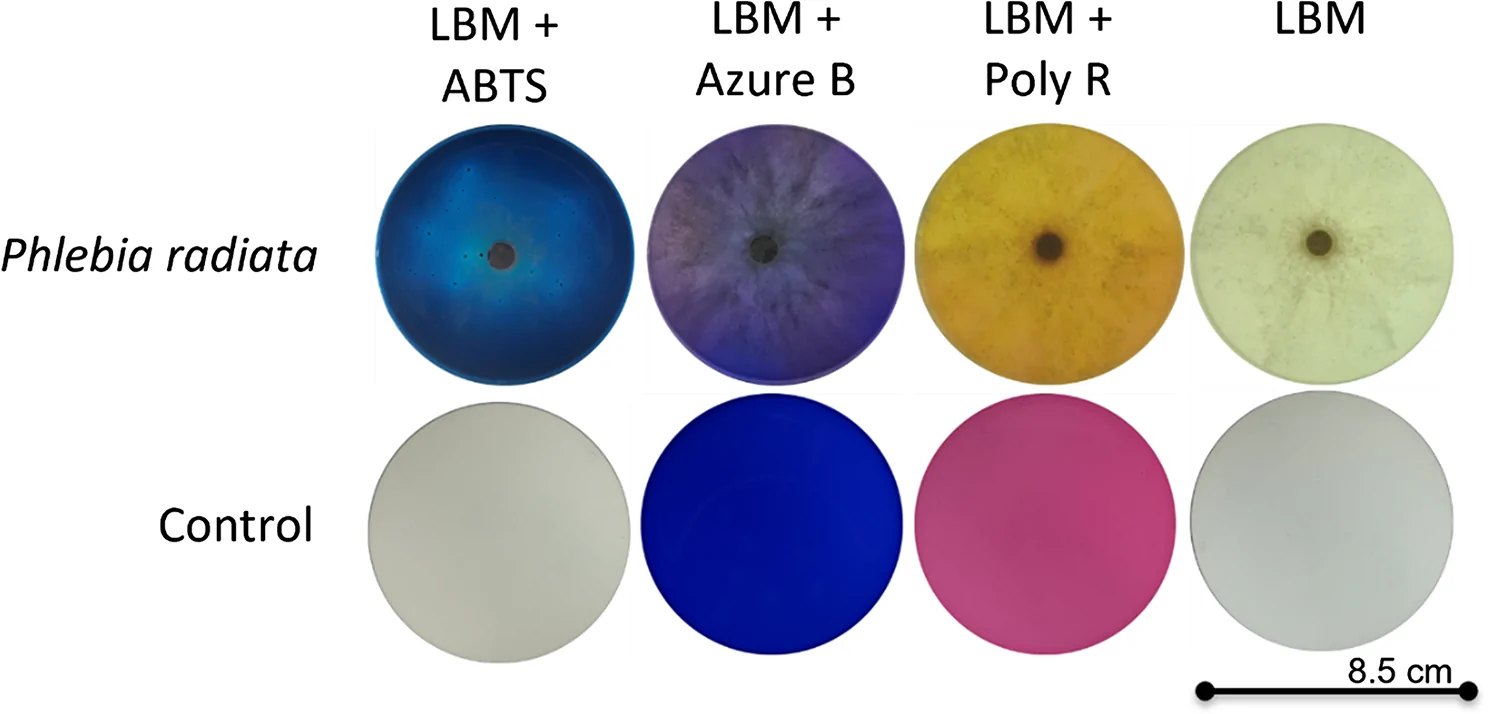
Qualitative assessment of ligninolytic enzyme activity in *Phlebia radiata*, on LBM agar. Blue–green staining of ABTS-containing plates reflects laccase activity, while decolorization of Azure B and Poly R-478 indicates peroxidase activity and/or laccase activity. Fungal growth was compared with controls on LBM agar without additives. Negative controls consisted of media supplemented with ABTS, Azure B, and Poly R-478 without fungal inoculation. Images were captured after ten days of incubation at 23 °C in darkness, using a lightbox

Preliminary cultivation experiments were performed in several liquid media with and without 1% wood powder to identify conditions supporting extracellular oxidative activity and metabolite production (Fig. S1-2). Based on these experiments, ½ soy medium supplemented with 1% wood powder was selected for subsequent cultivation and metabolite analysis. Given the central role of ligninolytic enzymes in fungal wood conversion, qualitative dye-based plate assays were first performed to assess extracellular oxidative activity in *P. radiata* IBWF 87241 using ABTS, Azure B, and Poly R-478 as substrates (Fig. 1). This qualitative assay was used as an initial profiling prior to subsequent quantitative liquid-culture experiments.

*On ABTS-containing LBM plates*,* colonies of P. radiata* IBWF 87241 *were surrounded by a distinct blue-green halo*,* indicating ABTS oxidation.* In contrast, only limited Azure B decolorization was observed in the immediate vicinity of fungal growth, while substantial decolorization of Poly R-478 occurred during the incubation period (Fig. 1). *Uninoculated control plates retained their initial appearance throughout the incubation period*,* and colony growth and morphology of P. radiata* IBWF 87241 *were comparable across the different dye-supplemented media*,* as well as to the un-supplemented control.* These observations indicated extracellular oxidative activity under the tested conditions and supported subsequent quantitative analysis of extracellular enzyme activities during cultivation.

### Wood-containing cultures of *Phlebia radiata* show distinct extracellular chromatographic profiles and accumulation of aromatic α-hydroxy acids

Extracellular metabolites in cultures grown in ½ soy medium with or without 1% wood powder were monitored over a 28-day cultivation period using HPLC–DAD, LC–ESI–MS, and GC–EI–MS. Differences in HPLC–DAD chromatographic profiles recorded at 280 nm were detected between non-inoculated media controls and cultures inoculated with *P. radiata* (Fig. S3). Two prominent peaks present in uninoculated ½ soy medium, with or without wood powder, were markedly reduced following fungal cultivation, while a low-intensity signal at approximately 11.2 min was observed in cultures containing both fungal biomass and wood powder. Because of its low intensity and the lack of an unambiguous structural assignment, this signal was not interpreted further.

**Fig. 2:**
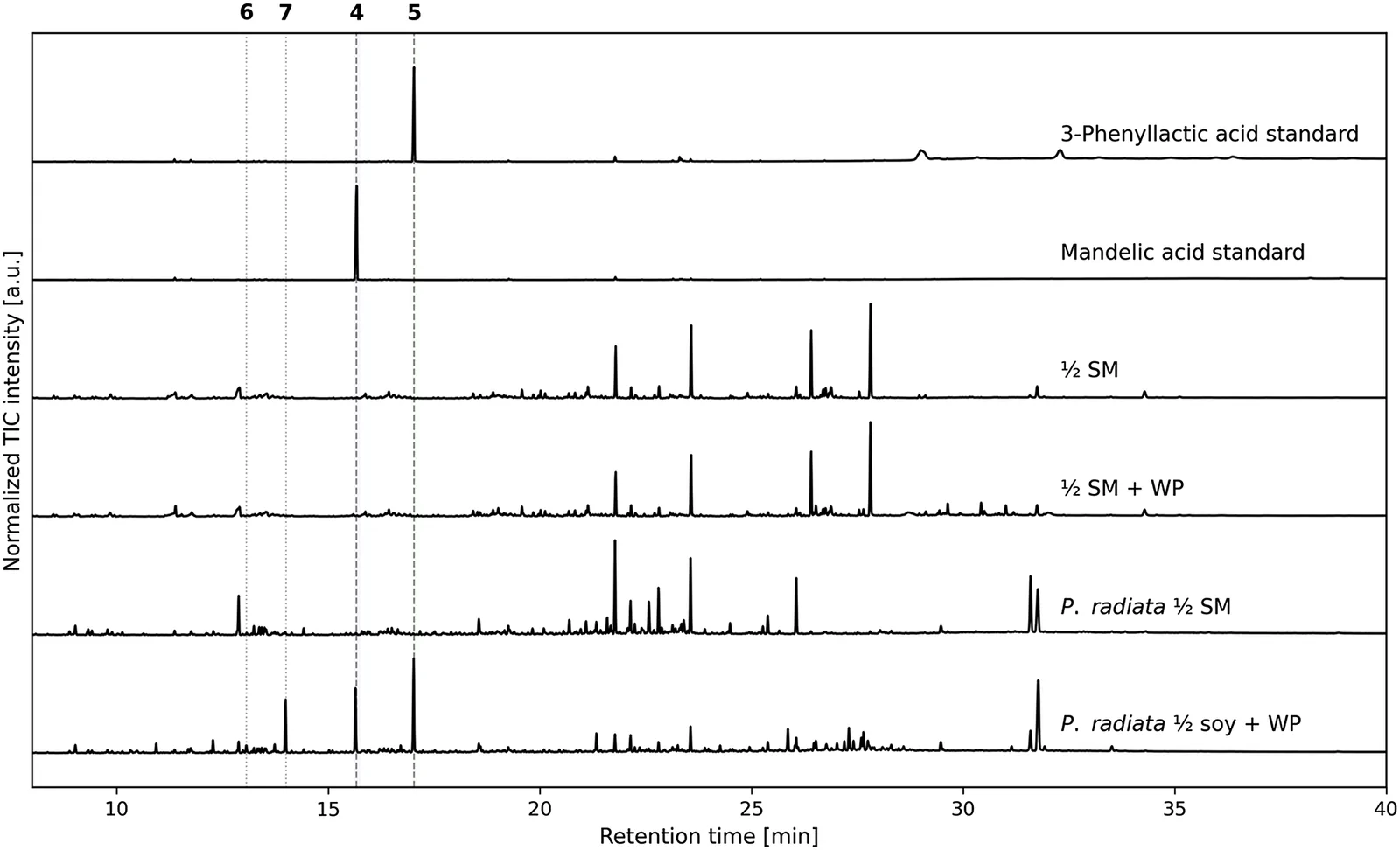
GC–EI–MS analysis of mandelic acid, 3-phenyllactic acid and additional condition-specific metabolites in culture extracts.GC–EI–MS total ion chromatograms (TICs) are shown for authentic standards of 3-phenyllactic acid and mandelic acid, uninoculated ½ soy medium (½ SM), uninoculated ½ SM supplemented with 1% wood powder (WP), *Phlebia radiata* cultivated in ½ SM, and *P. radiata* cultivated in ½ SM supplemented with 1% WP. Chromatograms were independently normalized and vertically offset for visualization. Vertical dashed lines indicate the retention times corresponding to mandelic acid (peak 4) and 3-phenyllactic acid (peak 5), as determined from authentic standards. Two additional peaks specific to the *P. radiata* + WP condition were detected at approximately 12.98 min (peak 6) and 13.99 min (peak 7) and were tentatively assigned by NIST library matching to niacin and pyrrole-2-carboxylic acid, respectively. Other prominent peaks were also detected in the corresponding control samples and were therefore not individually annotated. The y-axis represents normalized TIC intensity and does not represent absolute compound concentration

GC–EI–MS analysis confirmed the extracellular production of two compounds exclusively in cultures of *P. radiata* grown in ½ soy medium supplemented with 1% wood powder (Fig. 2). These compounds were absent from all uninoculated medium controls and from fungal cultures grown in the absence of wood powder, demonstrating that their production was observed only in cultures containing both fungal biomass and wood powder under the conditions tested. These compounds eluted at retention times of 15.6 min (peak 4) and 17.0 min (peak 5). Putative identification based on NIST library matching (match factors 96–97%) was confirmed by co-elution with authentic standards of mandelic acid and 3-phenyllactic acid under identical derivatization and GC conditions (Figs. S5-S6), with matching EI fragmentation patterns. The compounds were therefore unambiguously identified as mandelic acid and 3-phenyllactic acid. Two additional peaks specific to cultures containing both *P. radiata* and wood powder were detected at approximately 12.98 min (peak 6) and 13.99 min (peak 7) and were tentatively assigned by NIST library matching to niacin and pyrrole-2-carboxylic acid, respectively (Figs. S9-S12). Other prominent GC–EI–MS peaks were also present in the corresponding control samples and were therefore not considered condition-specific. Comparable production of both compounds was also observed in cultures supplemented with pine wood powder (preliminary data, not included in this study) indicating that aromatic α-hydroxy acid formation was not restricted to spruce as the lignocellulosic substrate. 

### Time-course analysis of extracellular oxidative enzyme activities during bioreactor cultivation of *Phlebia radiata*

**Fig. 3:**
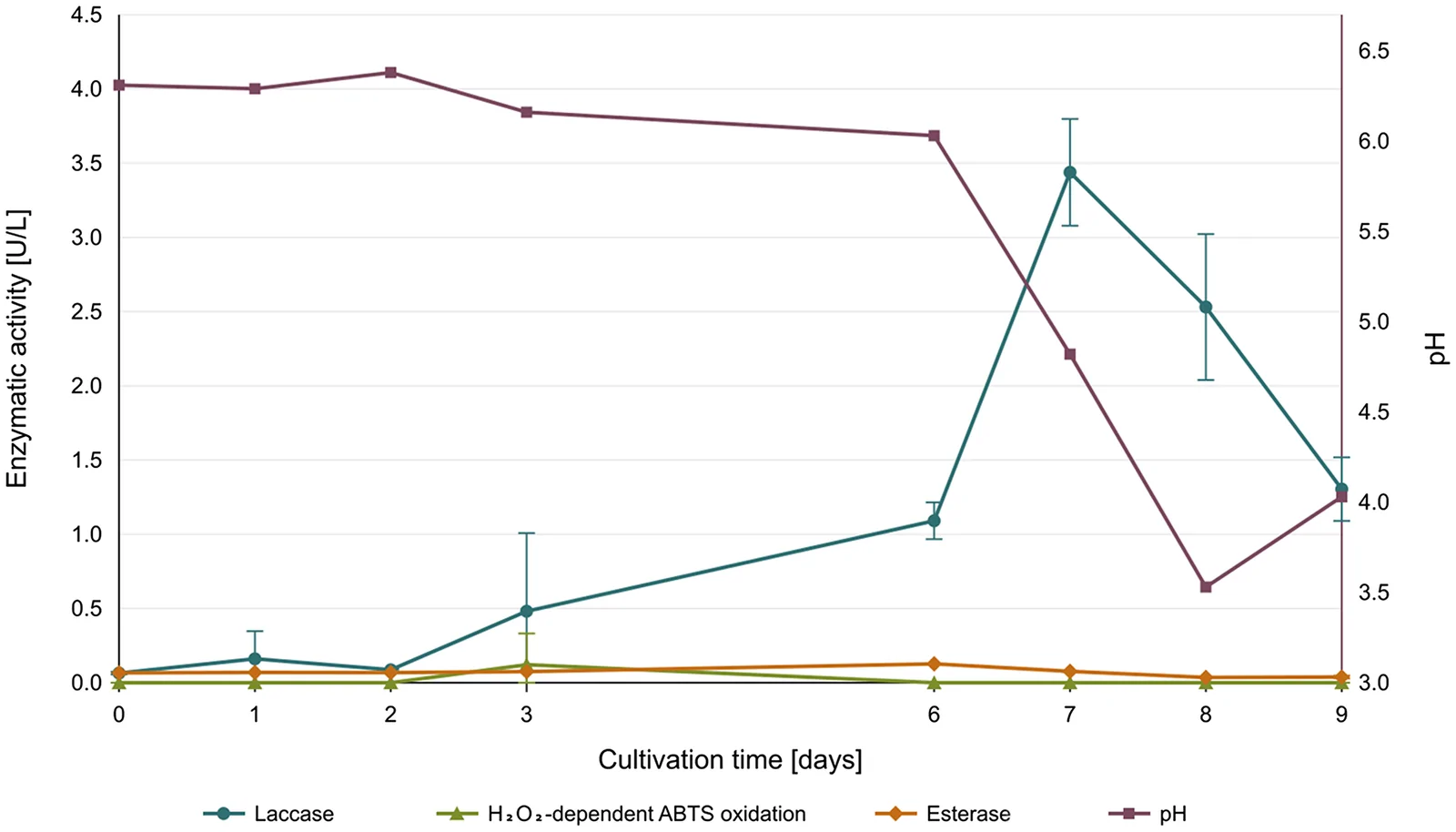
Time course of extracellular enzyme activities and culture pH during cultivation of *Phlebia radiata* in ½ soy medium supplemented with 1% wood powder. Extracellular laccase activity, H₂O₂-dependent ABTS oxidation, and esterase activity were measured in culture supernatants over a 9-day cultivation period and are plotted on the left y-axis (U L⁻¹). Enzyme activity measurements were performed in technical triplicate and are presented as mean ± SD; error bars therefore represent technical assay variability rather than biological variability. Error bars smaller than the plotted symbols may not be visible. The experiment represents a single 20 L stirred-tank bioreactor cultivation (*n* = 1 biological cultivation). Culture pH is shown on the secondary y-axis and represents a single measurement per sampling point. Laccase activity increased during cultivation, reaching a maximum on day 7 before declining, whereas H₂O₂-dependent ABTS oxidation remained low throughout the process under the assay conditions applied. Esterase activity remained comparatively low throughout cultivation

Following the identification of mandelic acid and 3-phenyllactic acid in small-scale cultivations, a 20 L stirred-tank bioreactor cultivation was performed to characterize extracellular enzyme dynamics and monitor process parameters during production (Fig. 3). Extracellular laccase activity, H₂O₂-dependent ABTS oxidation as an estimate of extracellular peroxidase activity, esterase activity, and culture pH were monitored throughout cultivation. Laccase activity increased during cultivation, reached a maximum around day 7, and then declined toward the end of the fermentation. H₂O₂-dependent ABTS oxidation remained low throughout the process under the assay conditions applied, whereas esterase activity was detected at comparatively low levels with a slight increase during the middle phase of cultivation. The pH of the culture remained near neutral during the early stages of cultivation and decreased during the later phase of fermentation.

### FTIR spectra indicate reproducible differences in recovered wood powder after cultivation with *Phlebia radiata*

**Fig. 4:**
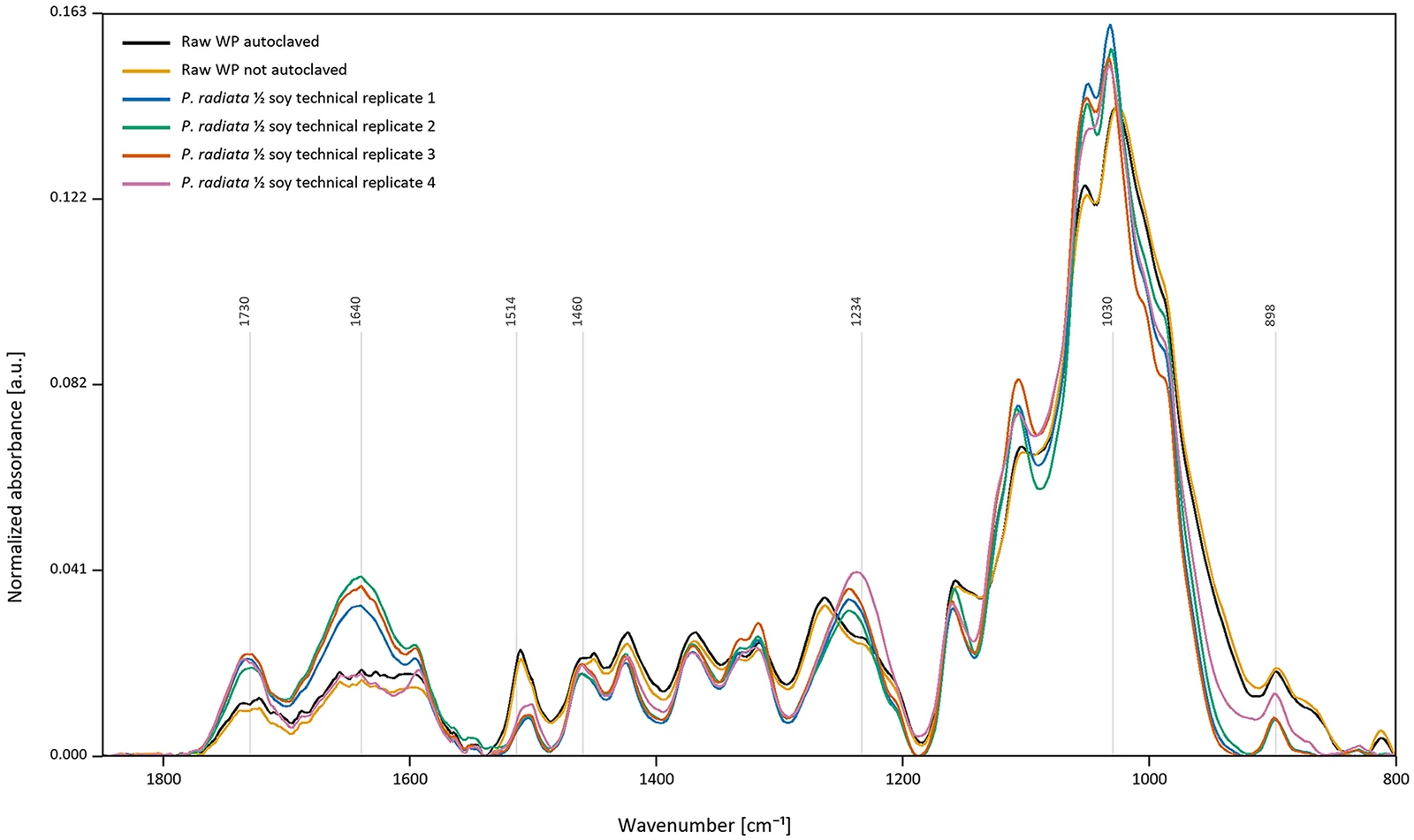
Comparison of normalized ATR-FTIR spectra of untreated and *Phlebia radiata*-treated wood powder. Vector-normalized ATR-FTIR spectra of raw autoclaved wood powder (grey), raw non-autoclaved wood powder (rosy brown), and recovered wood powder from four technical replicates of *Phlebia radiata* cultivated in ½ soy medium (blue, green, purple, and orange, respectively). Grey vertical lines indicate selected reference wavenumbers (1730, 1640, 1508, 1460, 1234, 1030, and 898 cm⁻¹). Spectra are shown to facilitate qualitative comparison of absorbance patterns between fungal-treated and untreated wood powder

Recovered wood powder obtained from the 20 L bioreactor cultivation was analysed by ATR-FTIR spectroscopy to investigate whether fungal cultivation was associated with reproducible differences in substrate absorbance patterns relative to untreated wood controls. The four technical replicates from fungal-treated samples displayed highly similar spectral profiles, indicating good reproducibility of the measurement and sample preparation. Compared with raw autoclaved and non-autoclaved wood powder, the recovered wood powder from *P. radiata* cultures displayed reproducible differences in several regions of the fingerprint spectrum (Fig. 4).

The most pronounced variation was observed for the vibration at 1640 cm⁻¹, where the fungal-treated samples showed higher relative absorbance than the untreated controls. This band is assigned to the C = O stretching in conjugated carbonyl groups and its persistence after successive washing and drying suggests that the observed difference was not solely attributable to residual water. This is an indication that fungal degradation of wood proceeds through oxidative cleavage and side chain oxidation of lignin leaving carbonyl rich residual lignin. This is also evident in the corresponding decrease in the aromatic skeletal vibration at 1508 cm⁻¹. Additional reproducible changes occurred near 1234 cm⁻¹ and across the C–O-dominated fingerprint envelope between approximately 1100 and 1000 cm⁻¹. Because this envelope comprises overlapping contributions from cellulose, hemicelluloses, and lignin, as well as vibrations influenced by hydrogen bonding, it cannot be attributed to a single wood component. Within this region, the feature near 1160 cm⁻¹ is more specifically associated with asymmetric C–O–C stretching of glycosidic linkages, whereas the band near 1030 cm⁻¹ contains overlapping C–O and ether-related contributions from both polysaccharides and lignin. The spectra of autoclaved and non-autoclaved raw wood powder were broadly similar, indicating that the observed differences were more pronounced in the recovered wood powder after fungal cultivation than between the two untreated controls. Overall, the FTIR data indicate reproducible differences in absorbance patterns between recovered and untreated wood powder in regions assigned to lignin- and carbohydrate-associated functional groups. However, because FTIR provides band-level information rather than direct compound identification, these data should be interpreted as evidence for substrate-associated spectral alterations rather than as proof of specific chemical transformations.

### Quantification of mandelic acid and 3-phenyllactic acid titers in scale-up cultivation of *Phlebia radiata*

**Fig. 5:**
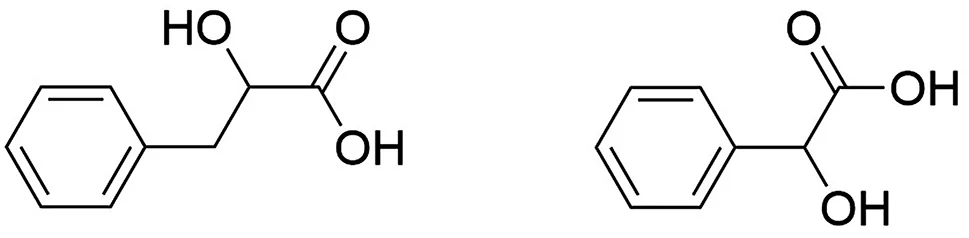
(left) 3-Phenyllactic acid (5) ((R, S)-2-hydroxy-3-phenylpropanoic acid). (right) Mandelic acid (4) ((R, S)-2-hydroxy-2-phenylacetic acid; C₈H₈O₃).Both contain an aromatic ring and an α-hydroxycarboxylic acid moiety and each possesses one stereogenic centre (structures shown as racemates unless stated). Structures are shown for the underivatized acids; in GC–MS analyses they were measured as their silylated derivatives. Compound numbers (4) and (5) correspond to the chromatographic peak labels assigned in the GC–MS analyses (Fig. 2 and Fig. S4)

Following identification of mandelic acid and 3-phenyllactic acid (Fig. 2) in small-scale cultivations, their production was further evaluated under controlled bioreactor conditions. To quantify product formation under controlled conditions, *P. radiata* was cultivated in a 20 L stirred-tank bioreactor under batch conditions using ½ soy medium supplemented with 1% wood powder. Culture samples were collected over time and analyzed by GC–EI–MS. Two prominent peaks corresponding to mandelic acid (TMS derivative; RT 15.624 min) and 3-phenyllactic acid (TMS derivative; RT 16.998 min) (Fig. 5) were detected in the day-7 total ion chromatogram (Fig. S4) and confirmed by comparison with authentic standards (Figs. S5–S6). Quantification using external calibration curves (Figs. S7-S8) yielded mean concentrations of 0.79 mg L⁻¹ mandelic acid and 4.11 mg L⁻¹ 3-phenyllactic acid in the day-7 bioreactor sample. The corresponding values obtained from three technical GC–EI–MS injections were 0.786 ± 0.042 mg L⁻¹ for mandelic acid and 4.107 ± 0.024 mg L⁻¹ for 3-phenyllactic acid (mean ± SD, *n* = 3 technical injections). These values were derived from a single bioreactor batch and represent preliminary production titers under the conditions applied. Based on the day-7 concentrations and a cultivation time of 168 h, the corresponding average volumetric productivities were 4.68 × 10⁻⁶ g L⁻¹ h⁻¹ for mandelic acid and 2.44 × 10⁻⁵ g L⁻¹ h⁻¹ for 3-phenyllactic acid. These titers correspond to estimated total amounts of 15.71 mg and 82.13 mg, respectively, in the 20 L cultivation. Details of the chromatogram, calibration, dilution scheme, and raw quantitative data are provided in Figs. S4–S8 and Tables S1–S3.

## Discussion

This study demonstrates that *P. radiata* IBWF 87241 is capable of producing mandelic acid and 3-phenyllactic acid extracellularly under defined lignocellulose-supplemented cultivation conditions, establishing a first quantitative production baseline for these aromatic α-hydroxy acids in a non-engineered white-rot fungal host. Across plate-based screening, bioreactor cultivation, FTIR analysis of recovered wood, and chromatographic profiling, wood addition consistently coincided with extracellular oxidative activity and a distinct extracellular metabolite profile. The results therefore show that extracellular oxidative activity and metabolite accumulation were both observed under lignocellulose-containing cultivation conditions.

During bioreactor cultivation, extracellular laccase activity increased steadily until day 7, whereas H₂O₂-dependent ABTS oxidation used as an estimate of extracellular peroxidase activity remained low and esterase activity was detected only at comparatively low levels. This pattern is consistent with previous reports describing substantial laccase production by *P. radiata* during growth on lignocellulosic substrates [24]. Enzyme secretion in *P. radiata* has been shown to vary across developmental stages rather than reflecting gene copy number alone [12, 24], indicating that fungi with similar CAZyme repertoires may display distinct extracellular oxidative phenotypes depending on cultivation conditions. Environmental factors including carbon-to-nitrogen ratio, copper availability, and oxidative stress have also been reported to influence the balance between laccase and peroxidase production [25], which may contribute to the enzyme activity profile observed during the present bioreactor cultivation.

FTIR analysis was used to assess structural changes in the solid wood fraction associated with extracellular oxidative activity. FTIR spectra were consistent with spectral patterns reported to accompany oxidative transformations of lignin, including assignments attributed to conjugated carbonyl groups or altered C = C stretching vibrations [26, 27]. While FTIR does not resolve specific bond cleavage events, the spectral changes are consistent with previously reported spectral patterns observed during fungal wood decay. Decreases in the aromatic skeletal band near ~ 1500–1515 cm⁻¹ are widely interpreted as indicative of lignin structural alteration, while increases in the ~ 1640–1650 cm⁻¹ region have been attributed to conjugated C = O groups formed during oxidative transformation of lignin side chains [26, 27]. Comparable changes have been reported for beech wood exposed to *Trametes versicolor*, where attenuation of aromatic vibration bands and increased intensity near ~ 1646 cm⁻¹ were associated with progressive oxidative modification of lignin during white-rot decay [26, 27]. Alterations in the ~ 1230 cm⁻¹ region have likewise been linked to changes in C–O stretching vibrations of syringyl units and lignin–carbohydrate complexes during fungal attack [26, 27].

Together, the extracellular enzyme activity profile, FTIR spectral differences observed in recovered wood, and production of mandelic acid and 3-phenyllactic acid document parallel observations made within the same lignocellulose-containing cultivation system. The present study does not establish a causal relationship between extracellular oxidative activity and aromatic α-hydroxy acid formation; these observations instead provide a framework for future mechanistic investigations integrating enzyme characterization, isotope tracing, and pathway analysis.

**Fig. 6:**
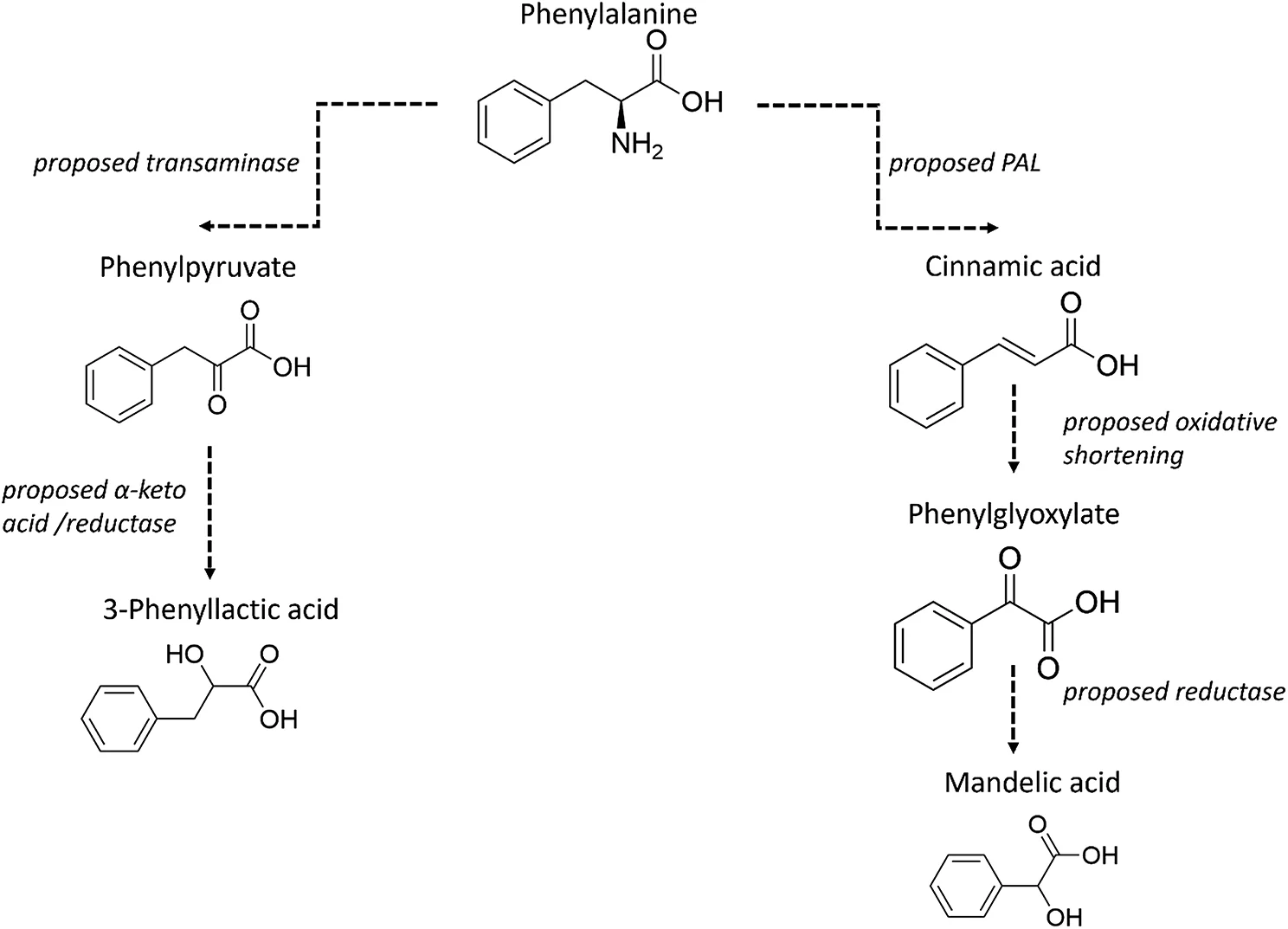
Proposed hypothetical pathways for the formation of 3-phenyllactic acid and mandelic acid in *Phlebia radiata*. The source of phenylalanine and other aromatic precursors in the present cultivation system remains unresolved and may include endogenous fungal biosynthesis, precursors derived from components of the soy/peptone-containing cultivation medium, wood-associated aromatic metabolism, or a combination of these sources. One possible route to 3-phenyllactic acid involves conversion of phenylalanine to phenylpyruvate by a putative aminotransferase followed by reduction of phenylpyruvate by an α-keto acid reductase. A second hypothetical route to mandelic acid involves deamination of phenylalanine to cinnamic acid, followed by oxidative side-chain shortening to phenylglyoxylate and subsequent reduction to mandelic acid. The pathways are inferred from previously described microbial aromatic acid and aromatic α-keto acid metabolism and were not experimentally validated in the present study

The biosynthetic origins of mandelic acid and 3-phenyllactic acid in *P. radiata* remain unknown. Two separate aspects must therefore be considered: the origin of the aromatic precursors and their subsequent conversion into the detected products. Aromatic precursors may arise from endogenous L-phenylalanine biosynthesis through the shikimate pathway, from phenylalanine or other aromatic precursors present in or released from medium components such as soy flour and Bacto peptone or, under wood-amended cultivation conditions, from aromatic compounds released during lignocellulose degradation. However, the relative contribution of these potential sources cannot be determined from the present data. Based on pathways characterized in bacteria and yeasts, 3-phenyllactic acid could be formed from L-phenylalanine through a two-step reaction sequence (Fig. 6). First, L-phenylalanine may be converted to phenylpyruvate by an aromatic aminotransferase. Phenylpyruvate could subsequently be reduced to 3-phenyllactic acid by an α-keto acid reductase or another 2-hydroxy acid dehydrogenase [28, 29]. Although this pathway is well established in several bacterial systems [29], its occurrence and the enzymes involved have not yet been demonstrated in *P. radiata*. For mandelic acid, which contains one fewer carbon in the side chain relative to phenylpyruvate, a direct reductive route from phenylpyruvate is not chemically feasible. A more likely route proceeds via phenylglyoxylate [30]. The metabolic origin of phenylglyoxylate in *P. radiata* is currently unclear. It may arise from endogenous aromatic amino acid metabolism or from the transformation of lignin-derived aromatic compounds, such as cinnamate- or benzoate-related intermediates. The detection of mandelic acid under wood-amended cultivation conditions is compatible with the latter possibility but does not establish lignocellulose as its direct carbon source. Thus, the pathways presented in Fig. 6 represent testable hypotheses rather than confirmed biosynthetic routes in *P. radiata*. Their validation will require time-resolved metabolite analysis, isotope-tracer experiments, identification of candidate genes and enzymes, and biochemical confirmation of the proposed reaction steps [28–30].

These compounds have been reported in microbial metabolic routes involving aromatic α-keto acid intermediates derived from amino acid metabolism [28, 30–32]. Their accumulation under wood-associated conditions therefore indicates an association between wood supplementation and extracellular aromatic metabolite accumulation in *P. radiata*, without resolving the source of the contributing precursors. The observation that mandelic acid and 3-phenyllactic acid were detected only in cultures containing both fungal biomass and wood powder demonstrates an association between wood addition and product accumulation; however, the aromatic precursors may originate from endogenous fungal metabolism, components of the soy/peptone-containing medium, wood-associated aromatic metabolism, or a combination of these sources. Distinguishing these possibilities will require isotope-tracer experiments using defined labelled precursor sources under appropriately controlled cultivation conditions. The present data demonstrate an association between oxidative cultivation conditions and α-hydroxy aromatic acid accumulation rather than a resolved biosynthetic pathway.

The titers obtained here (MA 0.79 mg/L; 3-PLA 4.11 mg/L) are substantially lower than the highest titers reported for engineered microbial systems (Table 1). Reported values in the literature span fundamentally different process types, including precursor-fed whole-cell biotransformations and fermentations in which the product is synthesized from a carbon source such as glucose or glycerol [28, 29, 31, 33–35]. These systems are therefore not directly comparable to one another or to the present cultivation. For 3-phenyllactic acid, non-engineered *Lactobacillus* sp. SK007 has been reported to produce 2.42 g/L in batch fermentation, whereas precursor-fed fed-batch cultivation with phenylpyruvic acid reached 17.38 g/L [35]. More extensively engineered Escherichia coli systems have reached substantially higher 3-PLA titers, including 52.89 g/L under optimized fed-batch conditions [34]. For mandelic acid, engineered *E. coli* strains have achieved titers ranging from sub-gram-per-litre levels to approximately 9.6 g/L depending on pathway design and cultivation strategy [29, 31, 33]. By contrast, the present titers were obtained with a non-engineered fungal host under wood-supplemented cultivation conditions, without deliberate addition of a defined aromatic precursor. Importantly, the origin of the aromatic precursor(s) leading to mandelic acid and 3-phenyllactic acid in *P. radiata* remains unresolved. The distinguishing feature of the present system is therefore not its titer, but the simultaneous formation of both aromatic α-hydroxy acids by a non-engineered white-rot fungus under wood-supplemented cultivation conditions [28, 29, 31, 33, 36, 37].

**Table 1:** Selected microbial systems for mandelic acid and 3-phenyllactic acid production compared with the present study

Organism / strain	Product	Production system	Carbon source / precursor	Engineering	Process	Reported titer	Ref.
*Lactobacillus* sp. SK007	3-PLA	Batch fermentation	Phenylpyruvic acid (5 g/L) + glucose	Non-engineered	Batch	2.42 g/L	[35]
*Lactobacillus* sp. SK007	3-PLA	Precursor-fed biotransformation	Phenylpyruvic acid + glucose	Non-engineered	3 L fed-batch	17.38 g/L	[35]
Coculture containing *P. pentosaceus* related isolate	3-PLA	Phenylalanine-supplemented fermentation	Phenylalanine + glucose	Non-engineered	Batch	1.225 g/L	[38]
Engineered *E. coli*	3-PLA	Fermentation	Glucose	Engineered	6 L fed-batch	52.89 g/L	[34]
Engineered *E. coli*	MA	Fermentation	Glucose	Engineered	Batch	0.74 g/L S-MA; 0.68 g/L R-MA	[29]
Engineered *E. coli*	(R)-MA	Fermentation	Glucose or glycerol	Engineered	Shake flask / coupled system	0.152–0.760 g/L	[31]
Engineered *E. coli*	MA	Fermentation	Glucose	Engineered	5 L HCDC	9.58 g/L	[33]
*P. radiata* IBWF 87241	MA + 3-PLA	Wood-supplemented cultivation	½ soy medium + 1% spruce WP; precursor origin unresolved	Non-engineered	20 L batch	MA 0.79 mg/L 3-PLA 4.11 mg/L	This study

Future work should focus on elucidating the biosynthetic pathways leading to mandelic acid and 3-phenyllactic acid formation in *P. radiata*. In particular, the observation that mandelic acid contains one carbon fewer than 3-phenyllactic acid and phenylpyruvate raises intriguing questions regarding potential oxidative side-chain shortening reactions or alternative aromatic metabolic routes that remain to be identified. Stable isotope tracing experiments combined with transcriptomic, proteomic, and targeted biochemical analyses will be essential to resolve the origin of these compounds and identify the enzymes involved. Identification of the responsible biosynthetic pathways would also provide a foundation for molecular engineering approaches, including targeted overexpression of key enzymes, elimination of competing pathways, and optimization of aromatic precursor flux to improve product titers. Beyond pathway engineering, process optimization strategies such as fed-batch cultivation and medium optimization may further enhance production. Given the established applications of mandelic acid in pharmaceutical synthesis and cosmetics, together with the antimicrobial properties of 3-phenyllactic acid, the ability of white-rot fungi to produce these compounds under lignocellulose containing cultivation conditions highlights their potential as sustainable biocatalysts for the generation of value-added aromatic chemicals within future lignocellulosic biorefinery concepts.

## Conclusion

In summary, this study establishes *P. radiata* IBWF 87241 as a non-engineered fungal production host for mandelic acid and 3-phenyllactic acid under lignocellulose-supplemented cultivation conditions. Preliminary bioreactor titers of 0.79 mg/L and 4.11 mg/L, respectively, represent the first quantitative production data for these compounds in a white-rot fungus and provide the basis for future bioprocess development and metabolic engineering strategies. Wood addition was consistently associated with elevated ABTS-oxidizing activity, reproducible FTIR spectral changes in recovered wood powder, and accumulation of mandelic acid and 3-phenyllactic acid under defined axenic conditions. Although the observed titers remain substantially below those reported for engineered microbial production systems, the findings demonstrate that lignocellulose supplementation is associated with altered extracellular metabolite profiles, including formation of aromatic α-hydroxy acids. Wood supplementation was associated with fungal production of aromatic α-hydroxy acids under the axenic batch conditions applied here. While the present titers remain far below industrial relevance, the study establishes a proof-of-concept baseline for future optimization strategies including transcriptomics-guided pathway analysis, cultivation optimization, and metabolic engineering approaches aimed at lignocellulosic aromatic bioconversion.

## Supplementary Information

Below is the link to the electronic supplementary material.

Supplementary Material 1

## Data Availability

No datasets were generated or analysed during the current study.
