# Supplementary material for: De novo production of mandelic- and 3-phenyllactic acid from lignocellulosic feedstock through Phlebia radiata IBWF-87241
Source: Microb Cell Fact. 2026 Oct 20;25:221. doi: 10.1186/s12934-026-03159-8 (PMC13700402; doi:10.1186/s12934-026-03159-8)
Supplement: Supplementary file 1 [file 12934_2026_3159_MOESM1_ESM.docx]

# *De novo* production of mandelic- and 3-phenyllactic acid from lignocellulosic feedstock through *Phlebia radiata* IBWF-87241

Fig. S1: Laccase activity of Phlebia radiata over time in different media. Cultivations were carried out in ten liquid culture conditions, including PDA, V8, ½ soy medium (½ SM), minimal medium (MM), and B5, each with or without 1 % (w/v) wood powder supplementation. Laccase activity (U/L) was measured over an 18-day cultivation period using the ABTS assay. One biological culture was analysed per condition (n = 1). Laccase activity measurements were performed in technical triplicate and are presented as mean ± SD. Error bars therefore represent variability among technical assay replicates rather than biological variability.

Fig. S2: Time course of pH, glucose concentration, and extracellular laccase activity of Phlebia radiata across five media with or without 1 % (w/v) wood powder supplementation. One biological culture was analysed per condition (n = 1). Extracellular laccase activity was measured using the ABTS assay in technical triplicate and is presented as mean ± SD. Error bars represent variability among technical assay replicates rather than biological variability. pH and glucose values represent individual measurements obtained from the respective culture samples and are shown without error bars.

Fig. S3: HPLC–DAD chromatograms (280 nm) of culture supernatants obtained after 28 days of cultivation in ½ soy medium (½ SM), ½ SM supplemented with 1 % wood powder (½ SM + WP), ½ SM inoculated with *P. radiata*, and ½ SM supplemented with 1 % wood powder and *P. radiata*. Chromatograms are displayed as stacked traces for clarity. Two prominent signals detected in non-inoculated media controls at a retention time of 6.5 and 7.8 min (peaks 1 and 2) were strongly reduced or absent in inoculated cultures. A low-intensity signal was observed at approximately 11.2 min in the P. radiata + WP condition; because of its low intensity and the lack of an unambiguous structural assignment, this signal was not interpreted further. The HPLC–DAD chromatograms were used for comparative profiling, and no compound identities were assigned from these data.

Fig. S4: GC–EI–MS total ion chromatogram (TIC) of the derivatized culture extract from the 20 L stirred-tank bioreactor cultivation of Phlebia radiata at day 7. The retention time window 14–19 min is shown in the inset. Peaks labelled 20 (RT 15.625 min) and 32 (RT 16.998 min) correspond to mandelic acid and 3-phenyllactic acid as their TMS derivatives, respectively, identified by co-elution with authentic standards (Figs. S5-S6). Mass spectra for both peaks are shown below the chromatogram. Peak numbers correspond to the peak report in Table S1.

Table S1: Complete peak report of the GC–EI–MS total ion chromatogram (TIC) obtained from the day-7 bioreactor culture extract shown in Fig. S4. Peak numbers correspond to those annotated in the chromatogram.

| **Peak#** | **R.Time** | **Area** | **Height** | **Name** |
| --- | --- | --- | --- | --- |
| 1 | 8.344 | 149798 | 83047 |  |
| 2 | 9.015 | 6239343 | 3333069 |  |
| 3 | 9.292 | 644760 | 357351 |  |
| 4 | 10.329 | 1513341 | 571282 |  |
| 5 | 10.921 | 303188 | 173500 |  |
| 6 | 11.026 | 130036 | 80140 |  |
| 7 | 11.360 | 482328 | 308871 |  |
| 8 | 11.749 | 412512 | 187350 |  |
| 9 | 12.201 | 2342848 | 1605532 |  |
| 10 | 12.269 | 207391 | 99276 |  |
| 11 | 12.425 | 163444 | 48985 |  |
| 12 | 12.833 | 249179 | 162703 |  |
| 13 | 12.867 | 370311 | 302459 |  |
| 14 | 13.317 | 9955237 | 6429543 |  |
| 15 | 13.514 | 319368 | 159494 |  |
| 16 | 13.799 | 324442 | 230209 |  |
| 17 | 15.082 | 2010648 | 1376674 |  |
| 18 | 15.280 | 164791 | 114910 |  |
| 19 | 15.375 | 186981 | 47094 |  |
| **20** | **15.624** | **957276** | **640958** | **Mandelic acid** |
| 21 | 15.717 | 151251 | 49890 |  |
| 22 | 15.800 | 149223 | 80992 |  |
| 23 | 15.836 | 394539 | 223274 |  |
| 24 | 15.915 | 2039288 | 1103829 |  |
| 25 | 16.046 | 215992 | 112053 |  |
| 26 | 16.191 | 178708 | 103259 |  |
| 27 | 16.267 | 175633 | 34004 |  |
| 28 | 16.391 | 238337 | 120943 |  |
| 29 | 16.483 | 263398 | 130088 |  |
| 30 | 16.707 | 238458 | 107793 |  |
| 31 | 16.850 | 152140 | 32861 |  |
| **32** | **16.998** | **5632595** | **3628516** | **3-Phenyllactic acid** |
| 33 | 17.541 | 155103 | 72523 |  |
| 35 | 17.990 | 297850 | 140246 |  |
| 36 | 18.242 | 165053 | 95780 |  |
| 37 | 18.364 | 1788684 | 971005 |  |
| 38 | 18.858 | 126138 | 79892 |  |
| 39 | 19.316 | 116740 | 81195 |  |
| 40 | 20.650 | 221353 | 117536 |  |
| 41 | 21.103 | 1474713 | 890029 |  |
| 42 | 21.759 | 3587062 | 2287474 |  |
| 43 | 22.125 | 157883 | 119164 |  |
| 44 | 23.541 | 2023511 | 1242835 |  |
| 45 | 25.908 | 250999 | 159599 |  |
| 46 | 30.415 | 8657569 | 4341088 |  |
| 47 | 30.675 | 287329 | 124503 |  |
| 48 | 31.734 | 444343 | 209276 |  |
| 49 | 32.369 | 717676 | 293687 |  |
| 50 | 34.280 | 561552 | 178123 |  |
| **Total** |  | **58,332,926** | **33,600,314** |  |

*Fig. S5: GC–MS analysis of the mandelic acid standard following derivatization using the same oximation/silylation protocol applied to culture samples (ethoxyamine hydrochloride followed by MSTFA). Total ion chromatogram (TIC) of the authentic mandelic acid standard showing a single dominant peak at a retention time of 15.670 min corresponding to mandelic acid as its 2TMS derivative. The inset mass spectrum (averaged over scans 920–922; RT 15.658–15.675 min) displays characteristic fragment ions at m/z 73 (base peak), 147, and 179, consistent with the fragmentation pattern of TMS-derivatized mandelic acid under electron ionization (EI) conditions. As mandelic acid contains no carbonyl group, the oximation step does not modify the analyte, and the observed derivative corresponds to the 2TMS form.*

Fig. S6: GC–MS analysis of the 3-phenyllactic acid standard following derivatization using the same oximation/silylation protocol applied to fungal samples (ethoxyamine hydrochloride followed by MSTFA). Total ion chromatogram (TIC) of the authentic 3-phenyllactic acid standard showing a dominant peak at a retention time of 17.023 min corresponding to the 2TMS derivative. The inset mass spectrum (averaged over scans 1083–1085; RT 17.017–17.033 min) exhibits characteristic fragment ions at m/z 73 (base peak), 147, and 193, consistent with the expected fragmentation pattern of TMS-derivatized 3-phenyllactic acid under electron ionization (EI) conditions. As 3-phenyllactic acid contains no carbonyl group, the oximation step does not modify the analyte, and the observed derivative corresponds to the 2TMS form. The prominent m/z 73 fragment confirms the presence of trimethylsilyl groups, indicating successful derivatization. This spectrum served as a reference for compound identification in fungal culture extracts.

Fig. S7: **External calibration curve for mandelic acid determined by GC–EI–MS. Integrated peak area at the quantification ion m/z 73 was plotted against the injected amount of mandelic acid. Five calibration levels were analyzed using three technical injections per level. Small open grey circles represent individual injections, while larger open black circles indicate the mean; error bars show ±SD. The black line represents the unweighted linear least-squares regression constrained through the origin. The resulting equation was y = 4.0196 × 10⁷x, with R² = 0.9994.**

Fig. S8: **External calibration curve for 3-phenyllactic acid determined by GC–EI–MS. Integrated peak area at the quantification ion m/z 73 was plotted against the injected amount of 3-phenyllactic acid. Five calibration levels were analyzed using three technical injections per level. Small open grey circles represent individual injections, while larger open black circles indicate the mean; error bars show ±SD. The black line represents the unweighted linear least-squares regression constrained through the origin. The resulting equation was y = 3.9048 × 10⁷x, with R² = 0.9994.**

Table S2: **Quantification of mandelic acid and 3-phenyllactic acid in the day-7 bioreactor extract by GC–EI–MS. Calibrated analyte amounts obtained from three technical injections of the same biological sample are shown together with mean ± SD and relative standard deviation (RSD). Quantification was performed using external calibration curves generated from derivatized authentic standards under identical analytical conditions**.

| Technical injection | Mandelic acid (µg) | 3-Phenyllactic acid (µg) |
| --- | --- | --- |
| Fermenter 7-1 | 0.0044 | 0.0233 |
| Fermenter 7-2 | 0.0042 | 0.02495 |
| Fermenter 7-3 | 0.0040 | 0.0236 |
| Mean ± SD | 0.0042 ± 0.0002 | 0.0234 ± 0.0001 |
| RSD (%) | 5.4206 | 0.6274 |

# Mandelic acid

**Mean calibrated amount:**

(0.0044 + 0.0042 + 0.0040) / 3 = 0.0042 µg

**Mass fraction in the crude extract:**

0.0042 µg / 0.5000 µg = **0.0084 = 0.8393% (w/w)**

**Amount of mandelic acid in the complete 4.1 mg crude extract:**

4.1000 mg × 0.0084 = **0.0344 mg = 34.4104 µg**

**Culture titer:**

0.0344 mg / 0.0500 L = **0.6882 mg L⁻¹**

**Rounded titer:** **0.69 mg L⁻¹**

**Technical-injection SD after conversion to culture titer:** **0.0373 mg L⁻¹**

**Estimated total amount in a 20 L cultivation:**

0.6882 mg L⁻¹ × 20 L = **13.7642 mg**

# 3-Phenyllactic acid

**Mean calibrated amount:**

(0.0233 + 0.0233 + 0.0236) / 3 = 0.0234 µg

**Mass fraction in the crude extract:**

0.0234 µg / 0.5000 µg = 0.0468 = 4.6781% (w/w)

Amount of 3-phenyllactic acid in the complete 4.1 mg crude extract:

4.1000 mg × 0.0468 = 0.1918 mg = 191.8019 µg

**Culture titer:**

0.1918 mg / 0.0500 L = 3.8360 mg L⁻¹

**Rounded titer: 3.84 mg L⁻¹**

**Technical-injection SD after conversion to culture titer: 0.0241 mg L⁻¹**

**Estimated total amount in a 20 L cultivation:**

3.8360 mg L⁻¹ × 20 L = 76.7208 mg

**Footnote**: A 10 µL aliquot of the crude extract solution (5 mg mL⁻¹), corresponding to 50 µg crude extract, was evaporated to dryness and derivatized to a final volume of 100 µL. The derivatized sample therefore corresponded to a crude-extract concentration of 0.5 mg mL⁻¹, and a 1 µL injection represented 0.5 µg crude extract. Because standards and samples were analysed under identical GC–EI–MS conditions, no correction for the split ratio was applied in the external-standard calculation.

Table S3: Final quantitative values for the 20 L fermenter.

| Parameter | Mandelic acid | 3-Phenyllactic acid |
| --- | --- | --- |
| Mean calibrated amount | 0.0042 ± 0.0002 µg | 0.0234 ± 0.0001 µg |
| Analyte fraction in crude extract | 0.8393% (w/w) | 4.6781% (w/w) |
| Analyte in 4.1 mg extract | 34.4104 ± 1.8652 µg | 191.8019 ± 1.2034 µg |
| Culture titer | 0.6882 ± 0.0373 mg L⁻¹ | 3.8360 ± 0.0241 mg L⁻¹ |
| Average volumetric productivity at day 7 | 4.0964 × 10⁻⁶ g L⁻¹ h⁻¹ | 2.2833 × 10⁻⁵ g L⁻¹ h⁻¹ |
| Rounded value for main text | 0.69 mg L⁻¹ | 3.84 mg L⁻¹ |
| Estimated total in 20 L | 13.7642 ± 0.7461 mg | 76.7208 ± 0.4814 mg |

The productivity calculation is:

- Mandelic acid: 0.6882 mg L⁻¹ ÷ 168 h ÷ 1,000 = 4.0964 × 10⁻⁶ g L⁻¹ h⁻¹

- 3-Phenyllactic acid: 3.8360 mg L⁻¹ ÷ 168 h ÷ 1,000 = 2.2833 × 10⁻⁵ g L⁻¹ h⁻¹

Fig. S9: **GC–EI–MS spectrum of the additional feature detected at RT 12.98 min in the** *Phlebia radiata* **+ wood culture extract.** EI mass spectrum of the feature eluting at 12.984 min in the derivatized culture extract of *P. radiata* IBWF 87241 grown in ½ soy medium supplemented with 1 % wood powder. The spectrum was averaged across RT 12.975–12.992 min. Major fragment ions were observed at m/z 180, 136, 78, and 106. The corresponding NIST library search is shown in Fig. S10.

Fig. S10: **NIST library search of the GC–EI–MS feature detected at RT 12.98 min.** Comparison of the EI mass spectrum of the feature detected at approximately 12.98 min in the *P. radiata* + wood culture extract with entries in the NIST14 mass spectral library. The highest-scoring match was **niacin (nicotinic acid) as its trimethylsilyl derivative**, with a similarity index (SI) of **88**. Because authentic-standard confirmation is not presented for this feature, the compound was considered tentatively assigned as niacin on the basis of library matching.

Fig. S11: **GC–EI–MS spectrum of the additional feature detected at RT 13.99 min in the** *Phlebia radiata* **+ wood culture extract.** EI mass spectrum of the feature eluting at 13.988 min in the derivatized culture extract of *P. radiata* IBWF 87241 grown in ½ soy medium supplemented with 1% wood powder. The spectrum was averaged across RT 13.983–14.000 min. Major fragment ions were observed at m/z 240, 73, 147, 166, 45, 196, and 255. The corresponding NIST library search is shown in Fig. S12.

Fig. S12: **NIST library search of the GC–EI–MS feature detected at RT 13.99 min.** Comparison of the EI mass spectrum of the feature detected at approximately 13.99 min in the P. radiata + wood culture extract with entries in the NIST14 mass spectral library. The highest-scoring match was **pyrrole-2-carboxylic acid as its 2TMS derivative**, with a similarity index (SI) of **89**. Because an authentic reference standard had not yet been analysed under identical conditions, the compound was considered **tentatively assigned as pyrrole-2-carboxylic acid** on the basis of library matching.

Table S4: Media components

| Minimal medium (MM) (pH 6.5) | 10 g Glucose (180.16 g/mol)  0.25 mL Biotin solution (0.01 %)  50 mL Nitrate salt solution (20x)  1 mL X‑Trace (1000x)  1 mL Thiamine dichloride solution (10 %) |
| --- | --- |
| MM + 1 % wood powder (WP) | 10 g WP  Add to 1 L MM |
| Potato-Dextrose Broth medium (PDA) (pH 5.6) | 26.5 g Potato-Dextrose |
| ½ Soy medium (½ SM) (pH 6.6) | 15 g Soy flour  15 g Maltose (342.90 g/mol)  5 g Bacto peptone |
| ½ SM + 1 % WP | 10 g WP  Add to 1 L ½ SM |
| Soy medium (SM) (pH 6.6) | 30 g Soy flour  15 g Maltose (342.90 g/mol)  5 g Bacto peptone |
| SM + 1 % WP | 10 g WP  Add to 1 L SM |
| B5 | 3.16 g Vitamin Gamborg  20 g Glucose (180.16 g/mol) |
| V8 | Vitafit Tomato-juice 200 ml |
